# An exercise trial for adults undergoing neoadjuvant chemoradiotherapy for rectal cancer proves not feasible: recommendations for future trials

**DOI:** 10.1186/s13063-020-04958-z

**Published:** 2021-01-06

**Authors:** Jennifer Brunet, Jenson Price, Céline Delluc

**Affiliations:** 1 School of Human Kinetics, University of Ottawa, 125 University Private, Montpetit Hall, Room 339, Ottawa, Ontario K1N 6N5 Canada; 2 Cancer Therapeutics Program, Ottawa Hospital Research Institute, The Ottawa Hospital, Ottawa, Ontario Canada; 3 Institut du savoir Montfort, Hôpital Montfort, Ottawa, Ontario Canada

**Keywords:** Recruitment, Retention, Feasibility, Methodology

## Abstract

**Background:**

The aim of this paper is to share the methodological problems of an unsuccessful prospective single-arm feasibility trial conducted to evaluate the safety and feasibility of a 12-week progressive exercise intervention for adults undergoing neoadjuvant chemoradiotherapy for rectal cancer, as well as offer recommendations for future trials.

**Methods:**

The initial plan was to recruit adults diagnosed with rectal cancer and scheduled for neoadjuvant chemoradiotherapy over a 12-month period. The exercise intervention was to consist of supervised exercise sessions delivered three times per week by a trained exercise specialist. Feasibility (i.e., recruitment, adherence, and compliance rates) and safety (i.e., adverse events) were to be assessed throughout the trial, and patient-reported and physical health outcomes were to be assessed pre- and post-intervention. After 8 months of open recruitment, we had been unable to successfully enroll patients into our trial. We therefore modified our eligibility criteria to increase the number of patients that could be recruited into our trial, and in turn increase our recruitment rate. We also amended our recruitment procedures to ensure we could reach patients who were either awaiting treatment, receiving treatment, or had completed treatments in the past 5 years. In doing so, we added a research objective, namely to determine the optimal timing of conducting an exercise intervention with adults diagnosed with rectal cancer (i.e., during neoadjuvant treatment, after surgery, during adjuvant treatment, or post-treatment).

**Results:**

Many problems continued to hinder the progress of our trial, particularly the low recruitment rate and the failure to enroll the required sample size that would make our results reliable.

**Conclusion:**

This led us to conclude that our trial was not feasible and that it is advisable to consider some elements carefully (e.g., recruitment strategies, communication, and trial location) before designing and conducting future trials. If one or more of these elements still proves to be problematic, trial results risk being compromised and alternative approaches should be considered.

**Trial registration:**

ClinicalTrials.gov NCT03049124. Registered on 02 September 2017

## Introduction

In 2020, colorectal cancer will be the 3rd leading cancer diagnosed in Canadian men and women [1]. Over 30% of these cancers are located in the rectum [2] and follow a different disease course and treatment plan than those located in the colon. The use of neoadjuvant chemoradiotherapy, which consists of either radiotherapy alone or in combination with chemotherapy, is a standard protocol used to treat most adults newly diagnosed with rectal cancer [3]. This protocol can increase the risk of developing short- and long-term side effects [4–6], which can impair quality of life and interfere with activities of daily living [7, 8]. Hence, survivorship programming dedicated to mitigating side effects and promoting quality of life has become a focus of cancer care.

Exercise is enthusiastically endorsed by several cancer agencies (e.g., American Cancer Society, Canadian Cancer Society, National Cancer Institute, Cancer Research UK, World Cancer Research Fund) as a complimentary therapy to help address survivorship challenges based on evidence that exercise is more beneficial than usual care in promoting quality of life for adults diagnosed with cancer [9, 10]. Although the health benefits of exercise for adults diagnosed with solid cancers are widely publicized, most of the evidence is based on studies with adults who have completed treatment for breast or prostate cancer [9–13]. Few studies have focused on adults diagnosed with colorectal cancer, and even fewer have focused solely on adults diagnosed with rectal cancer. Further, whilst the importance of exercise throughout the cancer continuum has been established [12, 13], studies with adults receiving neoadjuvant chemoradiotherapy are scarce as most have recruited adults post-treatment [14, 15]. Because adults receiving neoadjuvant chemoradiotherapy may experience side effects that can limit exercise tolerance, alter heart rate response to exercise, and increase the risk for exercise-related adverse events [4–6], establishing the safety, feasibility, and efficacy of exercise for adults diagnosed with rectal cancer who are receiving neoadjuvant chemoradiotherapy is crucial. In response, we designed a 12-week exercise intervention for adults receiving neoadjuvant chemoradiotherapy for rectal cancer and planned for a prospective single-arm feasibility trial to (1) determine if our intervention was feasible and safe, and (2) obtain initial estimates of the parameters of the main efficacy outcomes to inform sample size calculations for a future definitive trial. We hypothesized that (1) administering our exercise intervention during neoadjuvant chemoradiotherapy would be feasible and safe (i.e., no exercise-related adverse events would occur), (2) it would take < 1 year to recruit 14 patients, (3) dropout rate would be < 30%, (4) missing data would be < 5%, and (5) the average exercise session adherence rate would be > 70%. No hypothesis was formulated as to the anticipated parameter estimates for efficacy outcomes.

This article describes the lack of feasibility of our exercise trial for adults receiving neoadjuvant chemoradiotherapy for rectal cancer and outlines several barriers to the successful implementation of our trial. Although the trial was not successful, we feel that sharing our experience and offering suggestions for future trials provides useful information for improving methods for evaluating the feasibility, acceptability, and effects of exercise interventions for adults diagnosed with rectal cancer.

## Methods

Described below is the trial we attempted. Our initial goal was to determine if a 12-week exercise intervention offered to adults receiving neoadjuvant chemoradiotherapy for rectal cancer is feasible and safe. If successful, we had planned to expand it into a large-scale definitive trial to test the effects of our exercise intervention on patient-reported and physical health outcomes. Our protocol was approved by the Institutional Review Boards (IRB) at The Ottawa Hospital and the University of Ottawa.

### Criteria for inclusion

After consulting with an oncologist at The Ottawa Hospital, the eligibility criteria established in September 2017 were aimed at identifying adults diagnosed with rectal cancer who could safely participate in the exercise intervention. The criteria for inclusion were men and women (1) 18–85 years of age, (2) diagnosed with rectal cancer and scheduled for neoadjuvant chemoradiotherapy, (3) able to read/understand English, (4) ambulatory, (5) live < 50 km of the University of Ottawa, and (6) cleared to participate in the exercise intervention by a healthcare provider. Eligibility was established through a multi-step process involving screening by an oncologist and screening by trial staff.

### Criteria for exclusion

Initially, adults were to be excluded if they (1) had congestive heart failure, clinically significant aortic stenosis, history of cardiac arrest, use of a cardiac defibrillator, uncontrolled angina, uncontrolled arrhythmia, myocardial infarction, major heart surgery, stroke, or pulmonary embolism, (2) had uncontrolled hypertension (systolic blood pressure > 200 mmHg and/or diastolic blood pressure > 110 mmHg), (3) needed supplemental oxygen, (4) had severe arthritis (i.e., osteoarthritis or rheumatoid arthritis), (5) had a history of chest pain or severe shortness of breath either at rest or when engaging in physical activity, (6) had a hip fracture, hip replacement, or knee replacement in the past 6 months, (7) had impairments requiring mobility aids, (8) had stage IV cancer, (9) had a prior cancer diagnosis (excluding non-melanoma skin cancer), (10) were participating in another exercise trial, and/or (11) were unwilling/unable to give informed consent. In addition to these exclusion criteria, referring oncologists were to employ clinical judgment concerning patients’ safety; that is, if they judged the patient to be at high risk for adverse events or medical complications if they participated in the exercise intervention given their health status, they would not attempt to recruit the patient.

#### Changes to eligibility criteria

##### Change I

In 2017, the Canadian Society for Exercise Physiology released the *Get Active Questionnaire*—a self-administered, pre-exercise participation screening tool which replaced all previously Canadian Society for Exercise Physiology endorsed pre-exercise screening tools. Based on work completed by Thomas, Goodman, and Burr [16] showing that adults with high resting blood pressure (i.e., systolic blood pressure > 160 mmHg and diastolic blood pressure > 90 mmHg) should be regarded as higher risk (as cited in the *Get Active Questionnaire*), we amended our eligibility criterion around blood pressure to reduce the risk of adverse events (e.g., myocardial infarctions). Our revised exclusion criterion stated that patients who had systolic blood pressure > 160 mmHg and/or diastolic blood pressure > 90 mmHg were to be excluded.

##### Change II

Difficulties recruiting suitable patients within the diagnosis-to-treatment timeframe was one of the main reasons for non-recruitment. The length of time we had to recruit patients was short because the interval between diagnosis and initiation of neoadjuvant chemoradiotherapy was small. Thus, the length of time needed to be extended, leading us to open the trial to those diagnosed with rectal cancer who were either awaiting treatment, receiving treatment, or had completed treatments in the past 5 years.

### Procedures for patient recruitment

Initially, patients were to be recruited from The Ottawa Hospital. The following strategies were to be used to identify, recruit, and enroll patients. First, suitable patients treated at The Ottawa Hospital who had agreed to research contact through the institutional “permission to contact” registration process were to be approached. However, this strategy was dropped because it was not deemed feasible to identify potential patients due to the short time interval between diagnosis and treatment. Second, members of patients’ circle of care were to discuss the trial with them and obtain permission for trial staff to contact them to discuss the study. This process was overseen by a medical oncologist with an appointment at The Ottawa Hospital, whereby they would collect contact information for patients who consented from their colleagues and send it to trial staff who would then contact patients and review their eligibility.

#### Changes to recruitment strategies

##### Change III

Initially, patients learned about our trial through a member of their circle of care (generally their oncologist). Recognizing there are other channels that patients use to obtain information about ongoing trials, we added community-based recruitment strategies that included advertisements on social media websites (e.g., Facebook, Twitter, LinkedIn), online postings on bulletin boards/discussion groups and cancer-related websites (e.g., www.youngadultcancer.ca/community/blogs), and posters placed at locations that offer services to adults diagnosed with cancer in the Ottawa (Ontario) area (e.g., beauty stores, health clinics). We also had our community partners distribute posters to their clients and used word of mouth.

### Assessment procedures

Our plan was to have trial staff document the number of patients who (1) self-referred or were referred that met eligibility criteria (along with reasons for patients not meeting eligibility criteria), and (2) were enrolled over a 12-month period, as well as dropout rates, adherence, and percentage of missing data. Safety was to be assessed during the trial by having the exercise trainer record any adverse events during the exercise sessions using a standard form.

In addition, participants were to take part in assessments pre- and post-intervention (except for socio-demographic variables which were only to be assessed pre-intervention), which combined would take approximately 45 min to complete each time. The purpose was to collect data to describe the sample and to assess a range of outcomes (i.e., patient-reported and physical health outcomes), which were considered as primary outcomes or confounders for a future definitive trial. These assessments included a socio-demographic and medical questionnaire, the Functional Assessment of Cancer Therapy-Colorectal [17, 18], the Functional Assessment of Cancer Therapy-Cognitive Function [19], the Functional Assessment of Chronic Illness Therapy-Fatigue scale [20], the Positive and Negative Affect Schedule [21], and the Godin Leisure Time Exercise Questionnaire [22].

Finally, trial staff were to conduct physical assessments, which included resting heart rate measured using a Polar A300 heart rate monitor, resting blood pressure (mm Hg) measured in duplicate on the left arm using a blood pressure monitor (HealthSmart Digital Blood Pressure Monitor), body composition measured using a scale (Tanita TBF-310 GS), standing height measured using a Portable HR-200 height rod, aerobic capacity measured using the 6-min walk test [23, 24], and musculoskeletal strength measured using the combined grip strength of the right and left hands using a handheld dynamometer.

### Exercise intervention

The exercise intervention included three supervised exercise sessions per week for 12 weeks. Sessions were to be one-on-one and take place at the University of Ottawa. To support adherence to the exercise intervention, exercise sessions were to be scheduled around participants’ personal schedules. As such, sessions were offered on weekdays and weekends during regular working hours as well as early mornings and evenings. Sessions were to include a warm-up, aerobic training, strength training, and a cool-down component, and last between 60 and 75 min. The aerobic component was to consist of 30 min of individually prescribed exercise on a cycle ergometer or treadmill. Intensity was to be set at 60–75% of participants’ heart rate reserve and monitored using a Polar A300 heart rate monitor. The strength component was to consist of eight exercises that work the upper body, lower body, and core using machines and free weights. Participants were to start with one set of 10–12 repetitions per exercise and progress to two sets of 10–12 repetitions per exercise. Also, they were to start with light loads (50% of one-repetition maximum) and progress over time (75% of one-repetition maximum). The warm-up and cool-down components were to involve very light-intensity aerobic activity (< 60% of heart rate reserve) for 10 min. The intervention is in line with the cancer-specific recommendation provided by the American College of Sports Medicine [25, 26] and the Canadian Society for Exercise Physiology [27]. Sessions were to be stopped if any of the indications for exercise termination, as defined by the above organizations [25–27], were met. In this case, a note documenting the event was to be sent to the referring member of participants’ circle of care.

### Statistical analysis

Analyses were to be mainly descriptive. Patients’ socio-demographic and medical characteristics were to be summarized using descriptive statistics. Feasibility outcomes were to be reported as recruitment, retention, and adherence rates and percentage of missing data on assessed outcomes for the sample and their associated 95% confidence intervals. Patient-reported and physical health outcomes were to be summarized with means and standard deviations at each time point for continuous variables that were normally distributed and assessed by repeated measures analysis of variance to examine change from pre- to post-intervention. We were also to examine effect size (Cohen’s *d*) for change. Patient-reported and physical health outcomes were to be summarized with medians and interquartile ranges at each time point for continuous variables that were not normally distributed and assessed by Wilcoxon matched-pairs signed-rank tests to examine change from pre- to post-intervention. However, no conclusions about the efficacy of the exercise intervention were to be drawn from these results; rather, these analyses were purely to obtain parameter estimates to assist with the development of a future definitive trial.

## Results

**Fig. 1:**
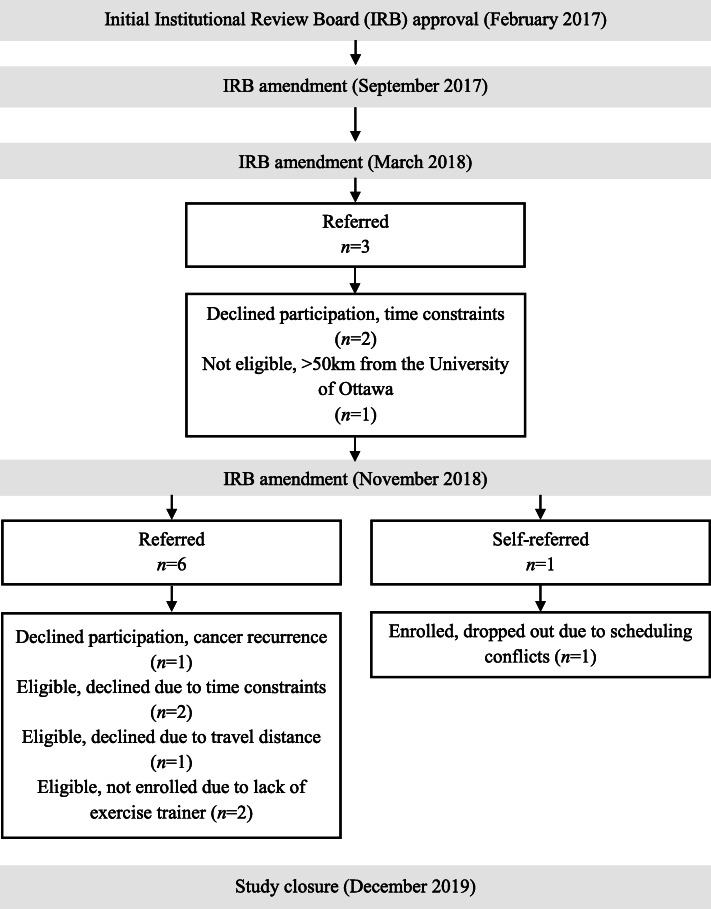
Summary of patient recruitment and enrollment

Figure 1 provides a summary of patient recruitment and enrollment. A total of 10 patients were referred (*n* = 9) or self-referred (*n* = 1) to the trial over a 21-month period, but 3 declined participation before being screened for the following reasons: time constraints (*n* = 2) and cancer recurrence (*n* = 1). Of the 7 patients who were screened, 6 were considered eligible; the other was not eligible because they lived > 50 km from the University of Ottawa. Of the 6 remaining eligible patients, 3 declined participation for the following reasons: time constraints (*n* = 2) and travel distance (*n* = 1). None of the remaining 3 eligible patients completed assessments or took part in the intervention because the trainer could not be retained (due to a lack of work; *n* = 2) and scheduling conflicts (*n* = 1), despite providing consent (*n* = 1).

## Discussion

The aims of our trial were to (1) determine if a 12-week supervised exercise intervention we developed for adults receiving neoadjuvant chemoradiotherapy for rectal cancer was feasible and safe, and (2) obtain initial estimates of the parameters of the main outcomes to inform sample size calculations for a future definitive trial. Unfortunately, it was hampered by a very low recruitment and enrollment rates. We encountered multiple challenges in our attempt to conduct this trial, which could be considered either protocol- or patient-related issues. We believe that these issues, along with suggestions for how to address them, should be shared with the scientific community to help bolster recruitment and retention in future exercise trials with adults diagnosed with rectal cancer.

### Protocol-related issues

A major challenge we encountered was the difficulty associated with identifying and accessing the pool of potential patients at The Ottawa Hospital who may have wanted to participate in our trial. Trial staff could not directly approach patients because of local policy that dictated the exclusion of the trial team from recruitment activities; rather, members of patients’ circle of care (generally their oncologist) were to identify eligible patients and refer them to the trial team via one oncologist. Despite sending frequent reminders to the coordinating oncologist and asking them to share these reminders with colleagues, recruitment may have suffered because it was not a team effort, such that one oncologist ended up having to coordinate everything rather than having multiple persons to alleviate the burden of recruitment. Perhaps having all members of patients’ circle of care provide a detailed explanation and/or have each send information to the trial team would help. Healthcare providers cite numerous constraints associated with discussing trial participation with adults diagnosed with cancer [28]. Similar constraints (e.g., time, knowledge) may have influenced members of patients’ circle of care ability to discuss our trial with patients, such that our trial took a secondary role to routine office visits. Having trial staff help recruit patients would have not only reduced their workload (and that of the coordinating oncologist), but would have provided expert knowledge of the trial processes and rationale. However, seeing that patients are more likely to participate in trials when their healthcare provider invites them to [29], it is possible that this would not have made a difference. Further exploration of this issue is required to determine if it would be advantageous to consider including trial staff as part of the recruitment team.

This was a single-site trial relying on sufficient numbers from The Ottawa Hospital and the surrounding area. Based on data provided by The Ottawa Hospital—the main hospital treating adults diagnosed with rectal cancer in Ottawa (Ontario)—the average number of adults who receive neoadjuvant chemoradiotherapy for rectal cancer each year is 35. In comparison to other prehabilitation single-site trials [30–33], this site may not have been a large enough pool from which to recruit willing adults with the specified socio-demographic and medical characteristics necessary for inclusion in this trial despite The Ottawa Hospital initially confirming having sufficient patients to meet trial timelines. Unfortunately, reasons for non-referral were not documented, making it impossible to determine if poor recruitment was associated with ineligibility, and if so, which inclusion/exclusion criteria were problematic. Nevertheless, the overestimation of the number of patients meeting eligibility criteria, also known as Lasagna’s law [34, 35], is a very common phenomenon in clinical trials and is especially challenging when eligible adults decline research participation. An obvious solution is to conduct a multi-site trial. To ensure each added site can contribute meaningfully to the conduct of the trial, an audit methodology, which can be used to determine a site’s ability to recruit to specific studies [36, 37], should be conducted. Specifically, an audit methodology utilizing surveys at each site of interest should be conducted to track the number of potentially eligible patients at a site over a pre-determined time period (e.g., 6 months) to better inform research planning by determining realistic recruitment goals for a site and to determine whether additional sites will be necessary to reach trial power.

The nature of the research may have had an influence on patient recruitment. First, an exercise trial could be harder to recruit for than an observational study because it requires greater commitment from patients in terms of time. This is evidenced by the main reason eligible patients offered for non-participation (i.e., time constraints, *n* = 4/10, 40%). Patients who are working or have other responsibilities may have been unable or unwilling to devote time to our trial. Moreover, the additional travel required to attend the supervised exercise sessions was also believed to be off-putting, especially for those who did not live close to the University of Ottawa. This is attributed to the broad catchment area The Ottawa Hospital serves, plus the large number of people who commute into the city for treatment from afar. Patients may be unlikely to enter an exercise trial if they anticipate it being difficult or if they doubt their ability to adhere to the protocol [38]. To overcome these barriers (i.e., time, travel), we would suggest that researchers clearly articulate the relevance of the trial to appropriate stakeholders: the patients and those who will help recruit patients into the trial (e.g., oncologists, nurses). The relevance of the trial needs to emphasize the possible benefits to present patients (despite trial burden), to future patients, to the healthcare system, and to society in general. In this case, it could be presented as an opportunity to access additional health services and health professionals. However, given that these barriers have been discussed by others as well [28, 39], it may be warranted to explore alternative methods of intervention delivery to decrease trial burden for patients. It may be useful to explore patients’ perspectives toward participating in an exercise intervention either at home (via teleconferencing or face-to-face with a trainer) or in the community, in which case forming collaborations with local communities to minimize patient travel would be necessary.

### Patient-related issues

Beyond our recruitment procedures, we may have also struggled to recruit patients into our trial because of our eligibility criteria. Our initial eligibility criteria were strict in selecting patients who were receiving neoadjuvant chemoradiotherapy for rectal cancer. We decided to recruit during this period because patients experience side effects that may limit exercise tolerance, alter heart rate response to exercise, and increase the risk for exercise-related adverse events [4–6]. However, the time interval between diagnosis and treatment may be stressful and laden with decisions [40], leaving many patients feeling overwhelmed [41]. Therefore, patients may not have consented to be contacted in the first place because they wished to focus on managing the numerous other changes to their life during this time. Nevertheless, others conducting prehabilitation trials with interventions delivered before surgery have had more success with recruitment [30–33]. For instance, Moug et al. [31] were able to recruit 78 adults to participate in their study exploring the feasibility of a randomized controlled trial for a walking group for adults receiving neoadjuvant chemoradiotherapy for rectal cancer. Over a 20-month period, of the 296 patients approached, 78 (26%) were eligible and 48 (62%) consented to participate in the trial. This recruitment success may have been associated with the recruitment method utilized and/or because of the intensity of exercise prescribed. Potential participants were approached by a colorectal cancer nurse specialist at the time of one of their surgical or oncological consultations and then followed up by a study team member, thus streamlining the recruitment process for patients. Consequently, whilst our trial demonstrates the difficulty encountered when recruiting from a population who is about to start treatment, it is possible that recruitment during this time may be bolstered by ensuring ease for patients and/or offering walking, as Moug et al. [31] did.

Also related to eligibility, there may have been situations where patients who could have participated in the exercise intervention may have been denied the opportunity to take part by the oncologist because they did not speak English. Indeed, patients who did not speak or understand English were not invited into the trial due to the absence of funding for interpreters and translation services. The influence of language on recruitment has been raised in other studies [29]; however, Grunfeld et al. [39] found that some study teams accommodated differences in language through family members and physicians who had the same first language as the patients. This possibility should be considered in future trials. Beyond eligibility criteria, it is possible that recruitment was hindered because of recruiters’ judgment due to assumptions about patients’ willingness to participate in an exercise intervention and the additional burden that would be needed to refer the patient [28, 29, 39]. An opt-out recruitment strategy, wherein patients are required to opt out of research teams contacting them about relevant research projects, compared to a conventional opt-in strategy, where clinically appropriate, could be a useful approach for addressing gatekeeping concerns and increasing recruitment to minimal risk trials [29, 42].

### Addressing challenges

Despite our best intentions, our trial was not feasible because of lower than expected recruitment and enrollment rates over a 21-month period. The trial was based on an estimation of 35 patients per year being treated at The Ottawa Hospital. Based on a referral target of 80%, we had planned to screen 28 patients for participation and to deliver the intervention to 14 patients (based on 50% meeting eligibility criteria) [43]. We received only 9 referrals (and 1 self-referral), and 4 did not pass the screening. The factors accounting for most of the exclusions were time and location constraints. After 6 months of active recruitment, we opened the inclusion period to any patient up to 5 years post-treatment, but this strategy failed to increase recruitment and thus enrollment was discontinued. Future trials may need to increase the number of referrals per year substantially, a rate that can only be achieved by large multi-site trials at national and international levels. In addition, feasibility may be enhanced by employing the following strategies.

#### Improving communication

It is necessary to evoke interest and curiosity among those who may be eligible to participate. As such, it is important to tailor communication about the trial to the target audience. Training recruiters on *how* to talk about the trial and ensuring they feel confident about their ability to explain the trial to patients may help. Also, explaining the importance of spending enough time to explain the trial process may be needed and being willing to answer hesitant patients’ questions about the trial is key. Whilst research is voluntary and therefore it is not possible to recruit patients who do not want to take part (and thus ensure one is not being coercive or embellish the benefits of the trial), there is a possibility that by engaging people in a conversation and clarifying misunderstandings and the trial relevance, patients might change their mind and be more open to exercise trials. Also, including adults diagnosed with cancer as active members of the research team and taking recruitment messages to a group of stakeholders can provide a unique perspective and ideas research teams may not have otherwise considered. There may be a need to use simpler or more commonly used terms to describe the trial, or there may be a need to include visuals about the intervention, what its potential benefits are, and why it is important. For example, it may not be enough to say, “exercise is good for you.” There may be a need to include photographs of what benefits may look like for patients receiving treatment for cancer.

#### Enhancing awareness

Lack of awareness of the trial can be a barrier in reaching recruitment goals. There are proactive approaches that should be considered to bolster recruitment. For trials with no or minimal funding for advertising (such as our trial), there are several avenues to explore. For example, asking community organizations that would be used by the trial demographic to share trial information on their websites, via newsletters, and by distributing posters in their waiting rooms could be beneficial. Researchers may also find it valuable to distribute posters to medical clinics, public libraries, recreation centers, coffee shops, grocery stores, and other stores that would be of interest to the trial demographic (e.g., stores selling ostomy care products). It is also possible to use online social media to promote the trial through “posts” on standalone trial pages and asking groups and pages to “post” on behalf of the research team. For trials that have a larger or substantial budget for advertising, there are additional online opportunities available. For example, Kaplan et al. [44] had success in creating an online matching tool that could be accessed by clicking on either the link embedded in the hospital website, searching for clinical trials using any internet search engine, or clicking on the trial’s advertisement (using a Google AdWords advertising campaign). In addition, researchers can purchase advertisements on online social media platforms to improve visibility of the trial.

#### Maintaining access

The literature, as well as our work, suggests that people are less willing to participate if there is a need to travel to complete assessments and/or trial components. Therefore, it is necessary that *where* the intervention and data collection take place be accommodating and flexible. Potential participants have their own responsibilities and challenges. One should ask themselves if either can take place face-to-face in the community or at other locations convenient to them (e.g., at their home). Indeed, incorporating community- or home-based options may make the trial more acceptable to patients. Thus, a recommendation is to consider offering exercise sessions via a hybrid exercise intervention (i.e., offering in-person instruction alongside online exercise sessions), in the community, or at participants’ homes (whenever possible) to avoid scheduling conflicts and to reduce the time burden of research participation. An alternative to hybrid or face-to-face exercise interventions is eHealth exercise interventions. Before doing this type of intervention, however, it is necessary to explore potential participants’ perspectives to understand how best to design eHealth exercise interventions so that they may have better success than the supervised exercise intervention we have described. Specifically, eHealth exercise interventions need to not only be consistent with quality standards of in-person exercise interventions, but address potential implementation barriers and determine how to manage possible adverse events. Additionally, eHealth exercise interventions should provide adequate support to enhance participants’ motivation and promote adherence as greater motivation and adherence to exercise interventions has been associated with improved health outcomes [45–47]. Based on recent reviews [48, 49], providing education, exercise instructions, training for self-regulation, goal setting, positive feedback, and reinforcement via real-time sessions and/or electronic means (e.g., emails) may enhance participants’ motivation and promote adherence to eHealth exercise interventions.

#### Maintain excitement about the trial

The oncologists may be patients’ only initial point of contact about the trial. Therefore, it is important that the oncologists helping with recruitment be enthusiastic about the trial. Indeed, those recruiting (oncologists or otherwise) need to maintain a positive attitude about the trial. There can be a lot of excitement built up about a trial when it first begins, but excitement can diminish as it wears on, especially long-term ones and if poor recruitment brings down morale. This can cause some to lose interest. Sending frequent reminders to maintain recruitment and offering incentives for referring oncologists (or otherwise) in terms of financial compensation and co-authorship could have a positive influence on recruitment.

## Conclusions

The primary aim of our trial was to (1) assess if a 12-week supervised exercise intervention we developed for adults receiving neoadjuvant chemoradiotherapy for rectal cancer was feasible and safe, and (2) obtain initial estimates of the parameters of the main outcomes to inform sample size calculations for a future definitive trial. Although we are unable to comment on the effects of the intervention given the intervention was not delivered, results demonstrate the difficulties encountered with recruiting and enrolling patients during treatment, and suggest our 12-week supervised exercise trial may not be feasible. We were surprised by this finding, although others have expressed similar challenges in this setting [50, 51]. Various factors may have influenced our ability to conduct our trial, including the nature of the research, study design, infrastructure, patients’ characteristics, and recruiters’ characteristics. Many of these are, in theory, amenable to modification through the development of systems to improve the identification and access of eligible patients, designing trials with reduced participant burden, and ensuring that recruiters have the motivation to recruit. Whilst patients may express interest, in reality, the decision to participate is multifaceted and requires them to consider the personal pros and cons of taking part. Those approached seemed to view the time commitment necessary to participate as a bigger barrier than the potential benefits of participating. We recommend the use of focus groups or interviews with potential participants to explore how to overcome this barrier, as well as to identify other possible barriers and facilitators to participating in exercise interventions. Moreover, a critical analysis of the methodological problems we encountered suggests that, before developing a trial, researchers should thoroughly evaluate recruitment means and the feasibility of reaching the entire population. We suggest obtaining information about why recruitment is low and holding team meetings with oncologists (or otherwise) to address issues. Also, we suggest considering different modes of delivery and data collection. In cases where patients are unwilling to travel, it is advisable to consider home/community-based, hybrid, or eHealth interventions. Either may have a higher likelihood of success and would be a reasonable target for additional research.

In addition, as mentioned above, chances are that one hospital alone did not have enough patients and collaborations with other sites may be needed. The plausibility and feasibility of the trial should be discussed with collaborating oncologists and sites. Initiating partnerships well before the trial begins will give some indication of the accrual rate and identify potential barriers. The feasibility of each site should be evaluated to ensure that there is an infrastructure available to support proper study conduct, including willing, experienced, and skilled trial staff; appropriate facilities to complete all trial activities; sufficient documented patient volume; and site investigators have the ability and willingness to comply with all trial procedures.

In closing, despite the focus on one site (i.e., The Ottawa Hospital) in the current trial, the issues identified are likely not unique to this location and therefore may be representative of other cities within this country and in other countries. As such, we hope that sharing our experience will assist others in the design and planning of future trials to treat side effects following a cancer diagnosis.

## Data Availability

Data sharing is not applicable to this article as no datasets were generated or analyzed.

## Abbreviation

IRB: Institutional Review Board
